# Gestational SARS-CoV-2 Infection in a Ugandan Birth Cohort: High Incidence, Mild Maternal Disease, and Evidence of Association with Transient Infant Stunting

**DOI:** 10.4269/ajtmh.23-0801

**Published:** 2024-09-17

**Authors:** Karen B. Jacobson, Katharina Röltgen, Brandon Lam, Patience Nayebare, Abel Kakuru, Jimmy Kizza, Miriam Aguti, Felistas Nankya, Jessica Briggs, Saki Takahashi, Bryan Greenhouse, Isabel Rodriguez-Barraquer, Kattria van der Ploeg, Jacob N. Wohlstadter, George B. Sigal, Michelle E. Roh, Joaniter I. Nankabirwa, Gloria Cuu, Stephanie L. Gaw, Philip J. Rosenthal, Moses R. Kamya, Isaac Ssewanyana, Grant Dorsey, Scott D. Boyd, Prasanna Jagannathan

**Affiliations:** ^1^ Department of Medicine, Stanford School of Medicine, Stanford, California, USA;; ^2^ Department of Pathology, Stanford School of Medicine, Stanford, California, USA;; ^3^ Department of Medical Parasitology and Infection Biology, Swiss Tropical and Public Health Institute, Allschwil, Switzerland;; ^4^ University of Basel, Basel, Switzerland;; ^5^ Infectious Diseases Research Collaboration, Kampala, Uganda;; ^6^ Department of Medicine, University of California, San Francisco (UCSF), San Francisco, California, USA;; ^7^ Meso Scale Diagnostics, LLC., Rockville, Maryland, USA;; ^8^ Department of Epidemiology and Biostatistics, UCSF, San Francisco, California, USA;; ^9^ Department of Internal Medicine, Makerere University College of Health Sciences, Kampala, Uganda;; ^10^ Department of Obstetrics, Gynecology & Reproductive Sciences, Division of Maternal-Fetal Medicine, UCSF, San Francisco, California, USA

## Abstract

Many questions remain about the prevalence and effects of SARS-CoV-2 infection in malaria-endemic African countries like Uganda, particularly in vulnerable groups such as pregnant women. We describe SARS-CoV-2 immunoglobulin (Ig)G and IgM antibody responses and clinical outcomes in mother-infant dyads enrolled in malaria chemoprevention trials in Uganda. From December 2020–February 2022, among 400 unvaccinated pregnant women enrolled at 12–20 weeks gestation and followed through delivery, 128 (32%) were seronegative for anti-SARS-CoV-2 IgG and IgM at enrollment and delivery, 80 (20%) were infected prior to or early in pregnancy, and 192 (48%) were infected or re-infected with SARS-CoV-2 during pregnancy. We observed preferential binding of plasma IgG to Wuhan-Hu-1-like antigens in individuals seroconverting up to early 2021, and to Delta variant antigens in a subset of individuals in mid-2021. Breadth of IgG binding to all variants improved over time, consistent with affinity maturation of the antibody response in the cohort. No women experienced severe respiratory illness during the study. SARS-CoV-2 infection in early pregnancy was associated with lower median length-for-age Z-score at age 3 months compared with no infection or late pregnancy infect (−1.54 versus −0.37 and −0.51, *P* = 0.009). These findings suggest that pregnant Ugandan women experienced high levels of SARS-CoV-2 infection without severe respiratory illness. Variant-specific serology testing demonstrated evidence of antibody affinity maturation at the population level. Early gestational SARS-CoV-2 infection was associated with transient shorter stature in early infancy. Further research should explore the significance of this finding and define targeted measures to prevent infection in pregnancy.

## INTRODUCTION

In contrast to soaring coronavirus disease 2019 (COVID-19) case numbers during pandemic waves in North America and Europe, malaria-endemic regions of Africa such as Uganda have reported relatively low numbers of COVID-19-related morbidity and death.^1^^,^^2^ Possible explanations include a younger population age structure with lower rates of comorbidities associated with more severe COVID-19,^3^ underreporting of the true disease burden in Africa,^4^ and potential differences in immunological background of populations, including cross-protective immunity from prior exposure to endemic coronaviruses or “trained” immunity from other pathogen exposures in African populations such as *P. falciparum.*^5^^,^^6^ Studies investigating immune responses to severe acute respiratory syndrome coronavirus 2 (SARS-CoV-2) infection in East African compared to North American or European populations and their effects in pregnancy are limited.

Given lack of widespread access to SARS-CoV-2 reverse-transcription polymerase chain reaction (RT-PCR) or rapid antigen testing in many African countries during the pandemic,^7^ testing of banked plasma specimens for the presence of anti-SARS-CoV-2 antibodies is a useful tool for understanding the epidemiology of SARS-CoV-2 infection in Africa.^8^ The most common targets for serological assays are the Spike (including the receptor-binding domain, RBD) and Nucleocapsid (N) proteins, as most individuals infected with SARS-CoV-2 develop antibodies to these antigens.^9^ Antibody cross-reactivity with other human coronavirus Spike and N antigens after SARS-CoV-2 infection has been reported in particular for SARS-CoV-1 and Middle East Respiratory Syndrome (MERS) coronavirus antigens.^10^ RBD mediates viral attachment and is therefore a dominant target of anti-SARS-CoV-2 neutralizing antibodies. During the pandemic, several SARS-CoV-2 variants with mutations in RBD (and other) genes have emerged and spread globally, facilitating evasion of neutralizing antibody responses elicited by COVID-19 vaccination or previous infection. We have reported previously that plasma samples collected from individuals after primary SARS-CoV-2 infection show characteristic serological profiles, with preferential binding to the RBD of the infecting variant, and that the breadth of antibody binding to other variants improves over time with ongoing affinity maturation.^10^ Thus, SARS-CoV-2 variant serotyping may be used to determine exposure to SARS-CoV-2 variants in epidemiological studies.

SARS-CoV-2 infection during pregnancy has been associated with more severe COVID-19 disease^11^ and preterm delivery.^12^^,^^13^ Increased fetal growth restriction (FGR) and lower birth weight was observed in some populations,^14^ but not others.^15^^,^^16^ Infection with Alpha or Delta variants may be associated with worse perinatal outcomes compared to infection with wildtype or Omicron variants.^17^^-^^19^ Although intrauterine and direct neonatal SARS-CoV-2 infection is rare,^20^ exposure to an inflammatory environment *in utero* may cause alterations in developmental pathways.^21^ There remains a lack of data from malaria-endemic settings in Africa related to effects of SARS-CoV-2 in pregnancy on birth outcomes, and data on longer term infant growth and development is even more limited.

Here, we retrospectively identify SARS-CoV-2 infections in a pregnancy cohort in eastern Uganda followed during the pandemic. We describe serologic responses to SARS-CoV-2 variants across pregnancy, and evaluated cross-reactivity to the Spike proteins of other human coronaviruses. We sought to determine whether SARS-CoV-2 infection in pregnant women was associated with symptoms and/or clinical outcomes in mothers and their infants.

## MATERIALS AND METHODS

### Pandemic pregnancy cohort.

**Figure 1.**
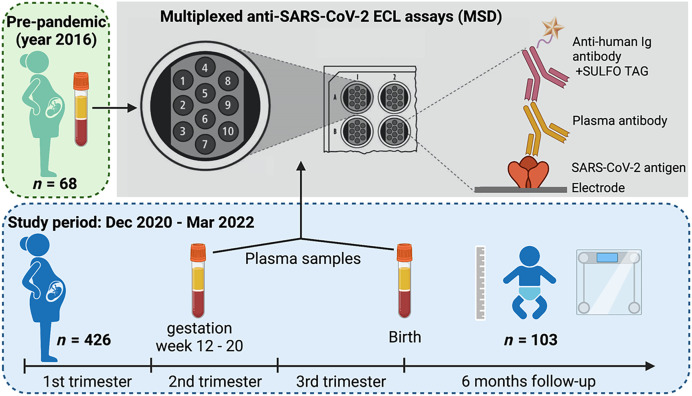
Study design. We tested stored plasma from *n* = 68 pregnant Ugandan women collected before the COVID-19 pandemic and *N* = 426 (26 vaccinated against SARS-CoV-2 prior to or during study participation, 400 unvaccinated) pregnant Ugandan women enrolled in a malaria chemoprevention trial during the COVID-19 pandemic. Paired samples collected at enrollment (early 2^nd^ trimester) and at delivery were tested for SARS-CoV-2 IgG and IgM on the MSD platform to assess for SARS-CoV-2 infection in pregnancy and associations with clinical and birth outcomes. Of 400 unvaccinated women, SARS-CoV-2 infection status in pregnancy was able to be determined for *n* = 320. Infant outcomes through 6 months of age were assessed in *n* = 103 infants (84 with maternal SARS-CoV-2 status known) followed to 6 months of age.

We leveraged data and samples from pregnant women and their infants enrolled in malaria chemoprevention trials ongoing during the COVID-19 pandemic (**Figure 1**). In the maternal trial, “Optimal chemopreventive regimens to prevent malaria and improve birth outcomes in Uganda” (NCT04336189), which began recruiting in December 2020, pregnant women were randomized to receive one of three monthly intermittent preventive treatment in pregnancy (IPTp) regimens: sulfadoxine-pyrimethamine, dihydroartemisinin-piperaquine, or sulfadoxine-pyrimethamine+dihydroartemisinin-piperaquine. Inclusion criteria were viable singleton pregnancy at 12–20 weeks gestation, not living with human immunodeficiency virus (HIV), age ≥16 years, resident of Busia District (Eastern Region of Uganda), and willingness to deliver in Masafu General Hospital. Exclusion criteria were long QT syndrome or active medical issue requiring inpatient treatment. At each clinic visit, a questionnaire was administered documenting presence or history of SARS-CoV-2 related symptoms (fever, fatigue/malaise, nausea, vomiting, diarrhea, cough, headache, rhinorrhea, abdominal pain, loss of taste/smell). Women were referred for SARS-CoV-2 PCR or rapid antigen testing at the discretion of medical staff and subject to availability of testing materials. Socioeconomic data was collected for each mother at a home visit. Plasma samples were collected at enrollment and at delivery. After delivery, placental tissue was evaluated by histopathology for evidence of placental malaria. In addition, birth outcomes such as spontaneous abortion, stillbirth, low birth weight, preterm birth, and small for gestational age were assessed.

Starting in October 2021, infants born to women enrolled in the maternal IPTp trial were enrolled in two separate studies. In the IMPACT (Infant malaria following IPTp with artemisinin-based combination therapy) observational study, infants were followed to age one year. In an additional infant trial, “Enhancing immunity to malaria in young children with effective chemoprevention” (NCT04978272), infants were randomized to receive IPT in childhood (IPTc) with monthly dihydroartemisinin-piperaquine up to age one or two years, or placebo, and followed to age four years. Inclusion criteria for both infant studies included infant’s mother enrolled in the maternal IPTp trial, resident of Busia district, and age 4–8 weeks at infant enrollment. Infants with an active medical problem requiring frequent medical attention were excluded.

Both women and infants enrolled in the studies received all medical care at the dedicated study clinic at Masafu General Hospital, were encouraged to come for unscheduled visits in the case of any febrile episode or other illness, and were tested for malaria parasites with blood smear at each unscheduled visit with fever. Routine assessments were conducted every four weeks at the study clinic with collection of clinical data including a symptom survey and blood by finger prick for the detection of malaria parasites by microscopy and quantitative PCR (mothers and infants), and recording of length and weight (infants).

From the maternal IPTp trial, all women delivering before March 2022 with available samples collected both at enrollment (12–20 weeks gestation) and at delivery (Figure 1) were identified. In this subset were 426 women with 852 paired plasma samples (collected at enrollment and at delivery) identified for testing.

### Plasma samples from pre-pandemic Ugandan study population.

Stored plasma samples collected in 2016 were obtained from 68 pregnant Ugandan women enrolled in a previous malaria chemoprevention trial at the same study site (NCT02793622).

### SARS-CoV-2 serology testing.

Blood was collected in standard blood collection tubes and transported to the immunology laboratory located on the campus of Tororo District Hospital where plasma was separated by centrifugation and stored at −80°C. Plasma samples were tested for SARS-CoV-2-specific antibodies using the Meso Scale Discovery (MSD) electrochemiluminescence (ECL) platform. Samples were heat-inactivated at 56°C for 30 minutes and tested in a 96-well plate format with MSD V-PLEX serology panels according to manufacturer’s instructions. V-PLEX COVID-19 Coronavirus Panel 3 kits were used to detect IgG antibodies to SARS-CoV-2 N, RBD, and Spike antigens and to Spike proteins of SARS-CoV-1, MERS-CoV, and other endemic HCoVs including HCoV-OC43, HCoV-HKU1, HCoV-NL63, and HCoV-229E in samples collected before and during the pandemic. V-PLEX SARS-CoV-2 Panel 8, 23, and 26 kits were used to determine IgG to different SARS-CoV-2 variant Spike and RBD antigens, including Wuhan-Hu-1, Alpha, Beta, Gamma, Delta, Eta, and Omicron in samples collected during the pandemic. We also used V-PLEX SARS-CoV-2 Panel 8 kits to determine IgM to SARS-CoV-2 N, RBD, and Spike antigens in samples collected before and during the pandemic. Samples were analyzed at a 1:5,000 dilution in MSD diluent. Coronavirus-specific antibodies were detected with anti-human IgG or IgM antibodies conjugated to SULFO-TAG ECL labels and read with a MESO QuickPlex SQ 120 instrument. Cutoff values for positive antibody test results were determined for each antigen by testing sera from Ugandan pre-pandemic pregnant women and were defined as the mean plus five standard deviations of the pre-pandemic antibody concentrations. IgG binding ratios for Wuhan-Hu-1 RBD and viral variant RBDs were calculated and plotted only for specimens that were above the highest RBD cutoff value for positive to avoid distortion of ratios by samples without specific binding. Each MSD plate contained duplicates of a 7-point calibration curve with serial dilution of a reference standard and a blank well. Calibration curves were used to calculate antibody unit (AU) concentrations by backfitting ECL signals measured for each sample to the curve.

To test the consistency of serological results for Wuhan Spike, a subset of samples were additionally tested using a Luminex (Austin, Texas) multiplex bead assay assessing total IgG responses to the Spike protein, as previously described.^8^

### Classification of coronavirus exposure status.

Samples were defined as positive for SARS-CoV-2 IgM if the AU concentration of Spike or RBD IgM antibodies was above the positive threshold as defined above, and positive for SARS-CoV-2 IgG if the AU concentration of Wuhan-Hu-1-specific Spike or RBD IgG, or any other variant Spike IgG, were above the positive threshold. Women who were negative for IgG and IgM at both enrollment and delivery were defined as uninfected with SARS-CoV-2. Women who were negative for both IgG and IgM at enrollment but positive for either or both IgG and IgM at delivery were defined as seroconverted and assumed to be infected during their study participation (i.e., in the second or third pregnancy trimester). Women with positive IgM (with or without positive IgG) at enrollment (between 12–20 weeks gestation), were classified as likely infected with SARS-CoV-2 during early pregnancy, given the expected decay of IgM within 60 days after infection.^10^ If a woman was IgG positive at enrollment and SARS-CoV-2 Spike IgG AU increased ≥4-fold between enrollment and delivery, they were considered to have a “boosted” response and likely re-infected during the study period.^22^ Women who seroconverted, boosted, or had early pregnancy exposure were classified as infected during pregnancy. Presumed variant of exposure was defined as the variant with the lowest Wuhan-Hu-1:variant RBD IgG AU ratio, with ratio <1, indicating a stronger response to the variant than to the early Wuhan-Hu-1 strain or other variants. 4-fold increases in IgG AU to the Spike proteins of OC43, HKU1, NL63, and 229E between enrollment and delivery were used as indication of infection with these other coronaviruses.

## STATISTICAL ANALYSES

Analyses were performed using R version 4.2.2 (https://www.r-project.org/). Differences in antibody concentrations to coronavirus antigens were tested using two-sided Wilcoxon rank sum tests. *P*-values <0.05 were considered statistically significant. In IgG Wuhan-Hu-1 to variant RBD ratio plots, a regression line shows the general trend of IgG binding to variants over time. Women’s characteristics and clinical outcomes were compared between those who were and were not infected with SARS-CoV-2 during pregnancy using Chi-square tests for categorical variables, Student’s t-tests for continuous variables if normally distributed, and Mann-Whitney U tests or Kruskal Wallis tests for continuous variables with non-normal distributions. To evaluate factors associated with infant stunting (length-for-age *Z* score <−2) at multiple time points during the first 24 weeks of life, we fit linear mixed effects regression models with random intercepts for each infant for the continuous outcomes of length- and weight-for-age *Z* score (per WHO growth standards^23^), with SARS-CoV-2 during early or late pregnancy as the primary exposure of interest, adjusting for measured factors that could additionally affect infant growth (maternal age, education, gravidity, maternal height, infant sex, infant age, and placental malaria). Models were also fit with interaction terms between SARS-CoV-2 infection and infant age, and between SARS-COV-2 infection and placental malaria.

### Study approval.

All study procedures were carried out with Institutional Review Board approval at Stanford University (Stanford, CA, USA; 61201, 62131), UCSF (San Francisco, CA, USA; 19-29105) and Infectious Diseases Research Collaboration (Kampala, Uganda; SBS-714, SBS-2021-39, SBS-2021-052).

## RESULTS

### High levels of SARS-CoV-2 infection were detected between late 2020 and early 2022.

**Figure 2.**
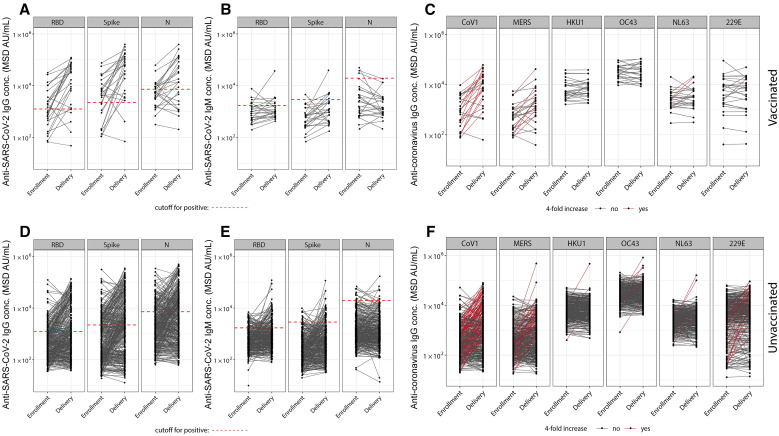
High levels of SARS-CoV-2 infection in Ugandan pregnancy cohort during COVID-19 pandemic. Anti-SARS-CoV-2 RBD, Spike, and N IgG (**A, D**) and IgM (**B, E**) antibody responses, and IgG antibody responses to the Spike protein of other human coronaviruses (**C, F**), are shown for samples taken at study enrollment and delivery from pregnant women in Uganda who were vaccinated (**A, B, C**) and unvaccinated (**D, E, F**) against SARS-CoV-2 prior to or during study participation. Paired samples are connected with black lines. Dashed red lines (**A, B, D, E**) indicate cutoff values for positive as determined by testing pre-pandemic samples from pregnant Ugandan women collected in 2016. Pre-pandemic background levels for anti-SARS-CoV-2 IgM antibodies were high compared to pandemic responses, particularly for anti-N. Lines connecting paired samples are red (**C, F**) for women who exhibited boosting of IgG levels by a factor of 4 or greater between enrollment and delivery.

We tested paired plasma samples from enrollment and delivery from 426 pregnant Ugandan women for the presence of anti-SARS-CoV-2 N, RBD, and Spike IgG (Figure 2A, 2D) and IgM (Figure 2B, 2E) antibodies using multiplexed MSD ECL assays. Many women showed increases in SARS-CoV-1, SARS-CoV-2, and MERS antibodies, and a smaller subset of women had at least 4-fold boosting of antibodies to the Spike protein of seasonal HCoVs NL63, HKU1, OC43 and 229E (*n* = 8, 5, 11, and 29, respectively; Figure 2C, 2F).

Of the 426 women, 26 (6.1%) reported being vaccinated and were excluded from further SARS-CoV-2 exposure analysis. Of 400 unvaccinated women, 220 (55.0%) were negative for anti-Spike and anti-RBD IgG and IgM at enrollment. Of these 220, 128 (58.2%) remained antibody negative at delivery and were presumed to have not been infected with SARS-CoV-2 in pregnancy, and 92 (41.8%) were anti-Spike and/or anti-RBD antibody positive at delivery and presumed to have experienced SARS-CoV-2 infection during pregnancy. Of the 180 who were positive for IgG and/or IgM at enrollment, 128 (71.1%) were positive for IgG only, 12 (6.7%) for IgM only, and 40 (22.2%) for both IgG and IgM. The 52 who were positive for IgM only or IgM and IgG were presumed infected with SARS-CoV-2 early in pregnancy prior to study enrollment. Of the 128 positive for only IgG at enrollment, 48 (37.5%) had antibodies boosted by a factor of 4 or greater at delivery and were presumed to have been re-infected during late pregnancy. Timing of infection could not be determined in 80 (20.0% of all unvaccinated women) with positive IgG and negative IgM at enrollment and no boosting at delivery, since infection may have occurred prior to pregnancy or early in pregnancy, and these women were excluded from clinical outcome analysis. Ultimately, we presumed that 128 women remained uninfected with SARS-CoV-2, and 192 were infected with SARS-CoV-2 during their pregnancy (Figure S1). There was high correlation between anti-Spike IgG as measured by MSD assays and Luminex testing performed for a subset of the samples (Figure S2).

### The breadth of SARS-CoV-2 variant RBD IgG binding improved over time.

**Figure 3.**
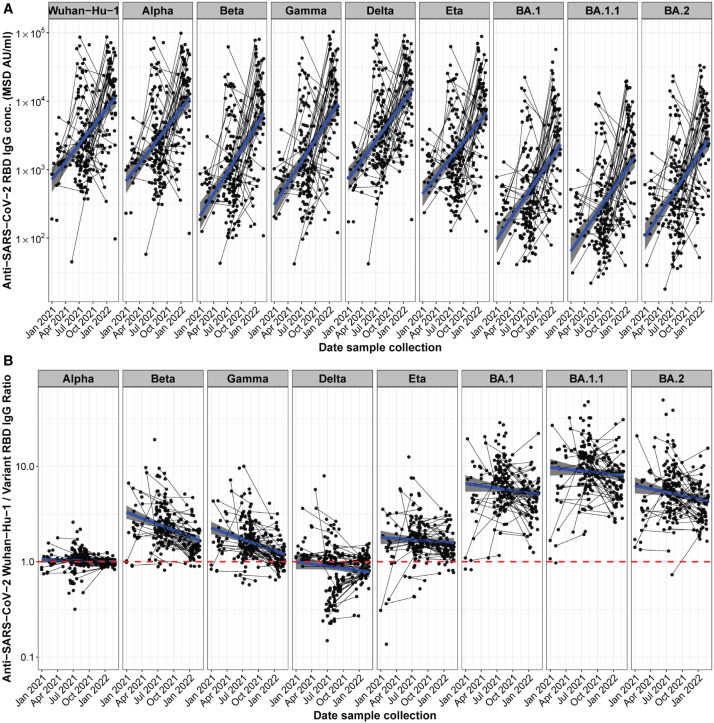
Exposure of a Ugandan pregnancy cohort to different SARS-CoV-2 variants. Anti-SARS-CoV-2 Wuhan-Hu-1 and viral variant RBD IgG responses in samples from pregnant women in Uganda between late 2020 and early 2022 are shown. Paired samples from study women at enrollment and delivery timepoints are connected with a black line. The blue locally estimated scatterplot smoothing (LOESS) regression line shows the trend of the development of the antibody response over time. (**A**) Anti-RBD IgG concentrations in MSD AU/mL. (**B**) Ratios of anti-Wuhan-Hu-1 to variant RBD IgG concentration. The red dashed line indicated a ratio of 1 in which the IgG variant response is equal to the anti-Wuhan-Hu-1 response; below the red line indicates preferential binding to the variant RBD, whereas above the red line indicates preferential binding to Wuhan-Hu-1 RBD.

We determined plasma IgG concentrations binding to the RBDs of Wuhan-Hu-1; five SARS-CoV-2 variants including Alpha, Beta, Gamma, Delta, and Eta; and three Omicron subvariants (BA.1, BA.1.1, and BA.2) using multiplexed MSD ECL assays (Figure 3A). We also compared the ratios of anti-RBD IgG concentrations for Wuhan-Hu-1 and other variants (Figure 3B). Based on the serological profile of RBD variant binding we identified putative Alpha, Delta, and Eta variant exposure mainly in early to mid-2021. IgG binding ratios, which showed a clear variant preference in the enrollment samples, changed over time toward equal binding to Wuhan-Hu-1 and variants in the delivery samples, indicating an increase in the breadth of the antibody response. Probable exposure to different Omicron subvariants in late 2021 and early 2022 was notable in that it was not accompanied by the expected preference of Omicron over Wuhan-Hu-1 RBD binding.

### Exposure to SARS-CoV-2 variants and reported symptoms in the pregnancy cohort.

Women reported cough or were diagnosed with an upper respiratory infection (URI) in 1201 of 7369 (16.3%, 95% CI 15.5–17.2) study visits over the study period. Reports of URIs and cough increased during the Delta wave in June 2021 to 24.5% (95% CI 10.8–28.6) and Omicron wave in December 2021 to 33.1% (95% CI 28.3–38.1). Report of fever did not similarly increase during these waves, however URI, fever, and cough all were increased during a period of high malaria incidence in February 2021 (Figure S3B). Cumulative numbers of SARS-CoV-2 infections were reflected in the increased seropositivity rate from less than 10% at enrollment in December 2020 to 100% in January 2022 (Figure S3C).

### Baseline characteristics and clinical manifestations associated with SARS-CoV-2 infection in pregnancy.

We next compared characteristics of women with and without SARS-CoV-2 infection during pregnancy (Table 1). There was no statistically significant difference in age, gravidity, maternal BMI at enrollment, education, or malaria incidence during pregnancy. There was a trend towards lower malaria parasitemia, as measured by qPCR at enrollment, in SARS-CoV-2-infected women compared to uninfected women (Table 1; 54.7% vs 64.8%; *P* = 0.07). However, a higher proportion of women living in modern houses (cement floors and walls) were infected with SARS-CoV-2 (Table 1; OR 1.72, 95% CI 1.05–2.85, *P* = 0.03) compared to those living in traditional houses (mud walls, thatch roof). On the other hand, a lower proportion of women in modern houses had malaria parasitemia at enrollment (OR 0.44, 95% CI 0.29–0.67, *P* <0.001).

**Table 1:** Maternal factors and clinical outcomes associated with SARS-CoV-2 infection in pregnancy

Variable	No SCV2 Infection during Pregnancy (*n* = 128)	SCV2 Infection during Pregnancy (*n* = 192)	Total (*N* = 320)	*P*-Value
Baseline Maternal Factors				
Age (years), median (IQR)	23 (19–29)	23 (19–28)	23 (19–29)	0.69
Multigravid, *n* (%)	90 (70.3%)	144 (75.0%)	234 (73.1%)	0.35
Mother’s BMI at enrollment, median (IQR)	21.7 (20.3–23.5)	22.1 (20.4–24.6)	21.9 (20.3–24.2)	0.11
O level education or higher, *n* (%)	43 (33.6%)	67 (34.9%)	110 (34.4%)	0.81
Modern house, *n* (%)	32 (25.0%)	70 (36.5%)	102 (31.9%)	0.03
Parasitemic by qPCR at enrollment visit, *n* (%)	83 (64.8%)	105 (54.7%)	188 (58.7%)	0.07
Malaria and SARS-CoV-2 Exposure				
Malaria Incidence per person year (95% CI)	0.42 (0.27–0.61)	0.55 (0.41–0.72)	0.50 (0.39–0.62)	0.28
Parasitemia prevalence (Positive qPCR tests/total number of tests, %)	260/1390 (18.7%)	408/2102 (19.4%)	668/3492 (19.3%)	0.60
Incidence of Reported Symptoms during Study Period Per Person Year (95% CI)				
Cough	4.3 (3.8–4.9)	4.1 (3.6–4.5)	4.2 (3.8–4.5)	0.46
Headache	3.9 (3.4–4.4)	3.1 (2.7–3.5)	3.4 (3.1–3.7)	0.01
Fatigue	0.91 (0.68–1.18)	0.63 (0.48–0.82)	0.74 (0.61–0.89)	0.065
Abdominal Pain	5.9 (5.3–6.6)	6.8 (6.3–7.4)	6.5 (6.0–6.9)	0.04
Diarrhea	0.45 (0.30–0.66)	0.67 (0.51–0.86)	0.58 (0.47–0.72)	0.11
Anorexia	0.18 (0.09–0.32)	0.30 (0.20–0.44)	0.26 (0.18–0.35)	0.17
Fever	3.4 (2.9–3.9)	3.0 (2.7–3.4)	3.2 (2.9–3.5)	0.25
Non-malarial fever	3.0 (2.5–3.4)	2.5 (2.1–2.8)	2.7 (2.4–2.9)	0.08
Delivery Outcomes				
Spontaneous abortion, *n* (%)	2 (1.6%)	4 (2.1%)	6 (1.9%)	0.743
Stillbirth, *n* (%)	3 (2.4%)	6 (3.2%)	9 (2.9%)	0.682
Preterm birth (<37 weeks), *n* (%)	5 (4.1%)	10 (5.5%)	15 (4.9%)	0.582
Gestational age at birth (weeks), median (IQR)	39.0 (38.0–40.0)	39.0 (38.0–40.0)	39.0 (38.0–40.0)	0.475
Newborn weight (g), median (IQR)	3000 (2800–3285)	3000 (2758–3300)	3000 (2770–3300)	0.944
Newborn length (cm), median (IQR)	47.0 (45.0–48.0)	47.0 (45.0–48.0)	47.0 (45.0–48.0)	0.646
Low birth weight (<2500 g), *n* (%)	11 (9.1%)	12 (6.6%)	23 (7.6%)	0.421
Small for gestational age (<10^th^ percentile), *n* (%)	21 (17.4%)	30 (16.5%)	51 (16.8%)	0.843
C-section delivery, *n* (%)	20 (16.0%)	35 (18.6%)	55 (17.6%)	0.551
Male infant, *n* (%)	54 (43.2%)	96 (50.8%)	150 (47.8%)	0.187
Mother’s WBC at delivery (cells/µl), median (IQR)	10470 (7117–12865)	10820 (8710–13395)	10650 (8260–13230)	0.106
Placental Malaria, *n* (%)	36 (30.0%)	45 (26.9%)	81 (28.2%)	0.571
Intervillous inflammation present, *n* (%)	21 (17.5%)	21 (12.6%)	42 (14.6%)	0.244

Infant Outcomes	Not Exposed in Pregnancy (*n* = 19)	Exposed in Pregnancy (*n* = 65)	Total (*N* = 84)	*P*-Value
Length in cm at 12 weeks of age, median (IQR)	59.6 (58.0–61.5)	58.0 (55.0–60.0)	58.0 (55.0–60.0)	0.008
Weight in kg at 12 weeks of age, median (IQR)	5.9 (5.3–6.2)	5.7 (5.3–6.3)	5.7 (5.3–6.3)	0.932
Stunting at 12 weeks of age, *n* (%)	0 (0.0%)	21 (32.3%)	21 (25.0%)	0.002
Underweight at 12 weeks of age, *n* (%)	0 (0.0%)	3 (4.6%)	3 (3.6%)	1.000

BMI = body mass index; IQR = interquartile range; qPCR = quantitative polymerase chain reaction for quantifying malaria parasite density; SCV2 = SARS-Cov-2; Stunting = length-for-age *Z *score less than -2 based on WHO Child Growth Standards; Underweight = weight-for-age *Z *score less than -2 based on WHO Child Growth Standards; WBC = white blood count. Malaria defined as fever+positive blood smear for malaria parasites. Placental malaria defined as malaria parasites or pigment on histology. *P*-values are reported for univariate logistic regression, chi-square tests of proportions for categorical variables, and Mann-Whitney *U* tests for continuous variables.

At the group level, increases in cough and URI corresponded to COVID-19 waves. However, individual symptom categories such as cough and fever were not associated with seroconversion Abdominal pain was more prevalent in SARS-CoV-2 infected women, but headache was more common in uninfected women (Table 1). Despite high rates of seroconversion for anti-SARS-CoV-2 antibodies during the study period, only three women were tested by rapid antigen test or PCR during the study period, and only one woman was diagnosed with COVID-19 by rapid antigen test. This woman had mild symptoms and no pregnancy complications. No women had severe respiratory illness requiring hospitalization or supplemental oxygen during study participation. One woman who seroconverted with presumed Delta infection died due to a surgical delivery complication unrelated to COVID-19. One woman with likely first trimester Alpha infection experienced transverse myelitis with unclear etiology; she was treated with steroids and recovered.

### SARS-CoV-2 infection during pregnancy was associated with stunting and lower length-for-age *Z *scores in early infancy.

We next examined whether SARS-CoV-2 infection during pregnancy was associated with adverse birth and infant outcomes. Though the proportions of spontaneous abortion, stillbirth, and preterm birth in infants born to infected mothers were marginally higher than uninfected mothers, these differences did not reach statistical significance (Table 1). There was no difference in median newborn weight or length, and though low birth weight (<2500 g), C-section delivery, placental malaria and intervillous inflammation on histology were marginally higher in infants born to SARS-CoV-2-uninfected mothers compared with infected mothers, again these differences did not reach statistical significance.

**Figure 4.**
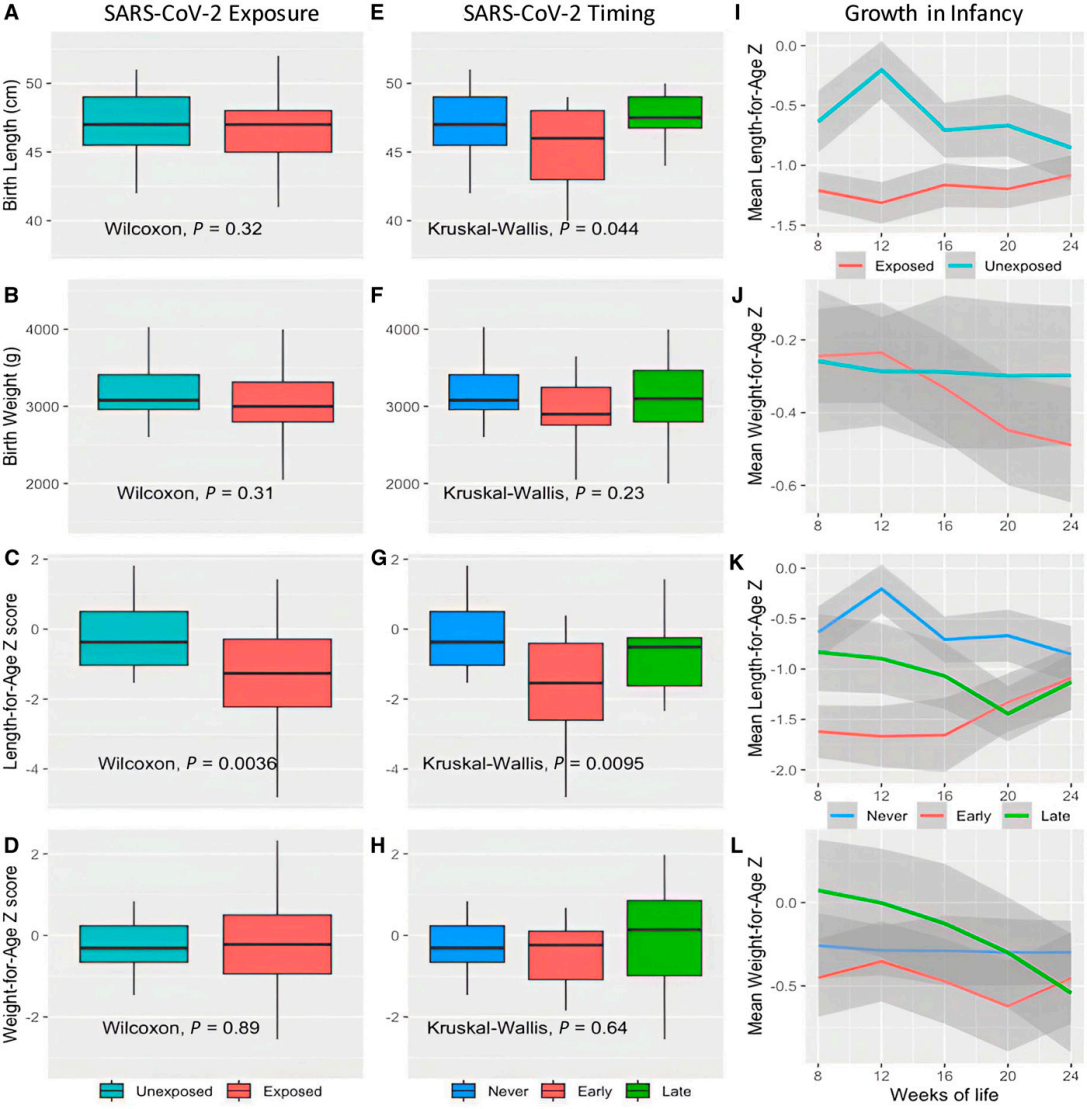
Gestational SARS-CoV-2 exposure and infant growth. Length- and Weight-for-age *Z *scores determined from WHO reference standards. Boxplots revealing infant length at birth, infant weight at birth, length-for-age *Z* score at age 12 weeks, and length-for-age *Z* score at age 12 weeks comparing SARS-CoV-2 exposed and unexposed (**A-D**) and timing of exposure (**E-H**). Panels I-L show mean and 95% CI (shaded region) of growth *Z *scores from 8 to 24 weeks of life comparing SARS-CoV-2 exposure (**I-J**) and timing of exposure (**K-L**). Exposed = exposed to SARS-CoV-2 during pregnancy as determined by seroconversion, positive Spike and/or RBD IgM at enrollment indicating recent infection, and/or boosting of IgG levels by a factor of 4 or greater between enrollment and delivery. Early infection = IgM positive at enrollment indicating recent infection in first trimester. Late infection = IgM and IgG negative at enrollment and IgM and/or IgG positive at delivery. Unexposed or never infected=remaining IgG and IgM negative at both enrollment and delivery.

However, among the 84 infants who were enrolled in the infant study and whose mothers’ SARS-CoV-2 seroconversion status in pregnancy was known, statistically significant associations were observed between SARS-CoV-2 exposure during pregnancy and infant length-for-age *Z* (LAZ) scores and stunting at age 12 weeks (Table 1). Birth length and LAZ scores were lower in infants born to mothers with early pregnancy SARS-CoV-2 infection (positive IgM at enrollment) compared to infants with uninfected mothers or mothers with late pregnancy SARS-CoV-2 infection (Figure 4). In linear mixed effects models with random intercepts for each participant, early pregnancy SARS-CoV-2 infection was associated with lower infant LAZ scores (Coef. = −1.10, 95% CI −1.81–−0.38, *P* = 0.003) in the first six months of life when adjusting for maternal and infant factors (Table 2).

**Table 2:** Linear mixed effects regression models to identify the effects of SARS-CoV-2 in pregnancy and other maternal and infant factors on length- and weight-for-age Z-scores through 24 weeks of life

Predictors	Length-for-Age *Z*-score				Weight-for-Age *Z*-score			
	Mean LAZ Difference	Standard Error	CI	*P*-Value	Mean WAZ Difference	Standard Error	CI	*P*-Value
SARS-CoV-2 (ref = uninfected)								
Early Pregnancy Infection	−1.10	0.37	−1.81 to −0.38	**0.003**	−0.82	4.17	−9.02–7.38	0.844
Late Pregnancy Infection	−0.15	0.40	−0.94–0.64	0.705	−0.39	0.37	−1.12–0.33	0.288
Gravidity	−0.23	0.11	−0.45 to −0.01	**0.038**	0.36	0.40	−0.44–1.16	0.374
Mother Achieved ≥O Level Education	0.14	0.33	−0.50–0.78	0.657	−0.07	0.11	−0.29–0.15	0.533
Height of Mother at Enrollment (cm)	0.04	0.03	−0.01–0.09	0.146	0.27	0.33	−0.38–0.91	0.422
Placental Malaria Present on Histology	−0.46	0.33	−1.11–0.18	0.159	0.01	0.03	−0.05–0.06	0.819
Female Gender	0.40	0.33	−0.24–1.05	0.217	−0.56	0.33	−1.21–0.10	0.096
Age of Infant (weeks)	0.01	0.01	−0.00–0.02	0.085	−0.01	0.00	−0.01 to −0.00	**0.001**

Length- and weight-for age *Z*-scores defined using WHO Child Growth standards. SARS-CoV-2 infection during early pregnancy defined as IgM positive at enrollment in second trimester. SARS-CoV-2 infection during late pregnancy defined as seronegative in second trimester and seropositive at delivery. BMI = body mass index. Bold indicates statically significant result.

To further investigate the trajectory of infant growth by gestational SARS-CoV-2 infection status and potential synergy of placental malaria, we fit additional linear mixed effects models for LAZ and weight-for-age *Z *(WAZ) scores in infancy with interaction terms between SARS-CoV-2 infection and infant age, and between SARS-COV-2 infection and placental malaria (Table S1). The interaction between early pregnancy SARS-CoV-2 infection and infant age had a small but significant positive association with the LAZ score (Coef. = 0.03, 95% CI 0.00-0.05, *P* = 0.030), indicating that infants born to mothers infected with SARS-CoV-2 early in pregnancy had increasing LAZ scores over time compared with infants born to uninfected mothers, in contrast to a decrease or no change in infant LAZ scores over time in infants born to mothers with late pregnancy SARS-CoV-2 infection compared with no infection (Coef. = −0.02, 95% CI −0.05–0.01, *P* = 0.243; Figure 4K). Conversely, WAZ scores decreased over time in infants born to SARS-CoV-2-infected mothers compared with uninfected mothers (Figure 4L). Interaction terms also revealed a potential synergistic negative effect (*P* <0.1) of late pregnancy SARS-CoV-2 infection and placental malaria on both LAZ (Coef. = −1.21, 95% CI −2.64–0.21, *P* = 0.095) and WAZ (Coef. = −1.31, 95% CI −2.82–0.21, *P* = 0.091) scores, but stratified analyses could not be performed due to low absolute numbers of women with both SARS-CoV-2 infection and placental malaria.

### Inferred variant SARS-CoV-2 infection and infant outcomes.

Using IgG binding ratios to different variants compared to Wuhan-Hu-1 to infer the viral variant responsible for a participant’s infection, we found that of the 168 with positive IgG at enrollment, 16 had predominant responses to Alpha variant Spike, 27 to Delta, 4 to Eta, and 1 to Gamma. Of 256 with positive IgG at delivery, 41 had predominant response to Alpha, 49 to Delta, and 15 to Gamma. Of the 320 women with SARS-CoV-2 infection status in pregnancy able to be determined, 31 were presumed infected with Alpha variant, 48 with Delta, and 11 with Gamma. We note that, due to varied enrollment dates, viral variant waves occurred at different times during pregnancy for each participant. Comparing infants of Alpha versus Delta infected mothers, there was no difference in birth outcomes (Table S2). Lower LAZ scores at age 12 weeks were more common in infants whose mothers were infected with Delta compared to Alpha variant in pregnancy (Figure S4).

## DISCUSSION

We observed high rates of SARS-CoV-2 exposure in this pregnancy cohort in eastern Uganda. We detected anti-Spike seropositivity rates similar to other serological surveys in East Africa, showing increases in population seropositivity to SARS-CoV-2 from 12% in late 2020 to nearly 100% by early 2022.^4^^,^^8^

Using precise internally controlled comparisons of polyclonal plasma IgG binding to different viral variant antigens, we identified preferential binding to Wuhan-Hu-1-like antigens in women infected through early 2021, with a shift to more women having preferential Alpha, Delta and other variant binding during later months of the pandemic. Thus, even if viral variant-specific nucleic acid testing or next generation sequencing was not available, serological testing has provided epidemiological data about viral variant exposures. Notably, the initial preferential binding of a patient’s plasma IgG to the likely variant infecting them was replaced over the course of months by broader binding to all viral variants in the panel of antigens tested. These data provide a population-level view of serological responses that are consistent with ongoing affinity maturation of IgG responses to SARS-CoV-2. Further analysis based on characterization of monoclonal antibodies derived from individual B cell clones detected over longitudinal sampling will be required to further characterize the mutational and affinity changes underlying these serological data. We note that responses to Omicron variants diverged from those to earlier antigenic variants in that women infected during Omicron waves had IgG responses that bound Wuhan-Hu-1 antigens better than Omicron antigens; potential explanations for this lack of preferential binding to the likely infecting viral variant include the reported lower stability of Omicron Spike antigens, or prior infection with earlier variants that imprinted the humoral response with non-Omicron specificity.^10^^,^^24^

Despite high rates of SARS-CoV-2 infection in this cohort, seroconversion was not associated with severe COVID-19 disease. This contrasts with large meta-analyses showing increased COVID-19 severity in pregnant women in many cohorts.^25^ Increased numbers of SARS-CoV-2 infections were reported in the general Ugandan population during Alpha, Delta, and Omicron waves (Figure S3A),^26^ and was temporally correlating with SARS-CoV-2 variant-specific serological ratios in our study cohort (Figure 3B). The Delta and Omicron surges in Uganda coincided with increased proportion of cough and upper respiratory infection-related visits, but not increased fever, in this cohort. Fever with COVID-19 may be less common in pregnant women than nonpregnant women, as has been reported in other African regions.^11^ SARS-CoV-2-infected women had fewer headaches and fevers compared to uninfected women, and no overall increase in cough or upper respiratory symptoms, consistent with findings in non-pregnant adults in the same setting,^8^ and suggesting that other common respiratory infections or non-infectious environmental pulmonary exposures may have accounted for symptoms in many individuals. Many women showed boosting of antibodies to SARS-CoV-1 and MERS, likely due to cross reactivity with SARS-CoV-2.^27^ A subset of women showed boosting of antibodies to endemic human coronaviruses NL63, HKU1, OC43, and 229E during the study period, and these viruses may have contributed to reported respiratory symptoms. No women developed severe respiratory disease consistent with COVID-19, although one woman experienced transverse myelitis, which has been reported in SARS-CoV-2,^28^ and recovered.

We did not find statistically significant associations between SARS-CoV-2 in pregnancy and birth outcomes including neonatal death, preterm birth, birth weight, birth length, or small-for-gestational-age. One possible explanation is that our sample size was insufficient to detect a difference in preterm birth or other birth outcomes. However, associations between gestational SARS-CoV-2 and preterm birth, pre-eclampsia, stillbirth, and low birth weight have been inconsistently reported in meta-analyses of observational clinical studies.^25^^,^^29^ Further, symptomatic or severe COVID-19 is associated with higher risk of preterm birth compared with asymptomatic or mild cases,^29^ so the mild COVID-19 disease observed in our cohort may have been less likely to be associated with significantly increased adverse birth outcomes.

Despite relatively small numbers of infants for whom follow up data was available, we noted differences in stunting in early infancy associated with maternal SARS-CoV-2 infection. To our knowledge, this is the first report of SARS-CoV-2 in pregnancy potentially affecting infant length.^30^ Many maternal infections are known to negatively impact fetal and infant weight including malaria,^31^ influenza,^32^ HIV^33^ and Zika,^34^ but there are scant data on any particular gestational infection affecting length of offspring. Historical data suggests that *in utero* influenza exposure could negatively impact stature through at least age eight years.^35^ In our data, stunting seemed greatest in infants born to mothers with positive IgM at enrollment, indicating early pregnancy infection, and in those with mothers infected with Delta variant, consistent with previous reports of Delta variant being more detrimental than other variants in pregnancy.^36^ Infections or inflammation early in pregnancy may disrupt limb development (established by eight weeks gestation) or growth *in utero*, disrupting the trajectory of growth and contributing to later length differences. This effect on infant length may be delayed and transient, since we observed that infants born to mothers with early pregnancy SARS-CoV-2 were similar in length to infants of uninfected mothers at birth, but had significantly lower LAZ scores within the first months of life. Conversely, LAZ scores of infants born to mothers with late pregnancy SARS-CoV-2 were initially similar to infants of uninfected mothers but had a relative decrease at 4–6 months of age. Infections in late pregnancy, particularly in the presence of placental malaria, may contribute more to weight differences, though these associations should be considered with caution due to the small number of women in this cohort with both SARS-CoV-2 infection and placental malaria. Further studies in larger cohorts are needed to better understand these potential associations.

This study’s limitations include reliance on retrospective clinical data from a malaria chemoprevention trial, reliance on antibody tests performed on banked specimens to determine SARS-CoV-2 exposure given a lack of nucleic acid testing in real time, and a relatively small sample size to examine associations between gestational SARS-CoV-2 and clinical outcomes. Despite the inherent difficulties in retrospective serological data, these data characterize a large and clinically well-defined pregnancy cohort in malaria-endemic East Africa during the first years of the SARS-CoV-2 pandemic where thus far infant follow up data has been limited. We were able to calibrate our positive cutoff using pre-pandemic samples from a geographically similar cohort. We have shown previously that MSD anti-Spike and anti-RBD assays are highly correlated with SARS-CoV-2 neutralization titers.^10^ The sero-epidemiologic analysis carried out here with multiplexed MSD ECL assays is likely scalable to other populations enabling detailed tracking of viral variant infections, the serological responses to them, and clinical outcomes, even in settings where resource limitations preclude nucleic acid testing or direct virus detection.

## Supplemental Materials

10.4269/ajtmh.23-0801 Supplemental Materials
