# Supplementary material for: Gestational SARS-CoV-2 Infection in a Ugandan Birth Cohort: High Incidence, Mild Maternal Disease, and Evidence of Association with Transient Infant Stunting
Source: Am J Trop Med Hyg. 2024 Sep 17;111(5):1107–17. doi: 10.4269/ajtmh.23-0801 (PMC11542540; doi:10.4269/ajtmh.23-0801)
Supplement: Supplemental Materials [file tpmd230801.SD1.pdf]

SUPPLEMENTAL MATERIAL

Table S1. Linear mixed effects regression models to identify the effects of SARS-CoV-2 in pregnancy and other maternal and infant factors on length- and weight-for-age Z scores through 24 weeks of life with interaction terms

	**Length-for-age Z score**				**Weight-for-age Z score**			
Predictors	*Mean LAZ difference*	*std. Error*	*CI*	*p*	*Mean WAZ difference*	*std. Error*	*CI*	*p*
**SARS-CoV-2 in pregnancy (Ref=Uninfected)**								
**Early infection**	-1.83	0.47	-2.75 – -0.91	**<0.001**	-0.18	0.47	-1.10 – 0.74	0.701
**Late infection**	0.71	0.55	-0.38 – 1.79	0.202	1.25	0.55	0.18 – 2.33	**0.023**
**Infant age (weeks)**	0.00	0.01	-0.02 – 0.02	0.776	-0.00	0.00	-0.01 – 0.01	0.861
**Placental malaria**	-0.45	0.47	-1.37 – 0.48	0.347	-0.16	0.51	-1.15 – 0.84	0.756
**Gravidity**	-0.20	0.11	-0.40 – 0.01	0.061	-0.04	0.11	-0.26 – 0.18	0.718
**Mother achieved ≥O level education**	0.36	0.31	-0.26 – 0.98	0.254	0.36	0.34	-0.30 – 1.02	0.281
**Height of mother at enrollment (cm)**	0.03	0.02	-0.02 – 0.07	0.300	-0.00	0.03	-0.05 – 0.05	0.925
**Infant sex (female)**	0.31	0.31	-0.29 – 0.92	0.309	0.05	0.33	-0.60 – 0.70	0.877
**Interaction**								
**Early infection x Infant age**	0.03	0.01	0.00 – 0.05	**0.030**	-0.01	0.01	-0.02 – 0.00	**0.065**
**Late infection x Infant age**	-0.02	0.02	-0.05 – 0.01	0.243	-0.02	0.01	-0.03 – -0.01	**0.007**
**Early infection x Placental malaria**	0.89	0.66	-0.40 – 2.18	0.174	-0.12	0.70	-1.50 – 1.27	0.869

**Late infection x Placental malaria**	-1.21	0.73	-2.64 – 0.21	**0.095**	-1.31	0.77	-2.82 – 0.21	**0.091**

Length-for-age (LAZ) and weight-for age (WAZ) Z scores defined using WHO Child Growth standards. SARS-CoV-2 infection during early pregnancy defined as IgM positive at enrollment in second trimester. SARS-CoV-2 infection during late pregnancy defined as seronegative in second trimester and seropositive at delivery. BMI=body mass index.

Table S2. Birth and infant outcomes by variant of infection

	Alpha (N=31)	Delta (N=48)	Total (N=79)	p value
**Spontaneous abortion**	1 (3.2%)	1 (2.1%)	2 (2.5%)	0.752
**Stillbirth**	0 (0.0%)	1 (2.1%)	1 (1.3%)	0.421
**Preterm birth**	4 (13.3%)	2 (4.3%)	6 (7.9%)	0.156
**Low Birth Weight**	1 (3.3%)	3 (6.5%)	4 (5.3%)	0.543
**Small for gestational age**	4 (13.3%)	6 (13.0%)	10 (13.2%)	0.971
**Mother's WBC at delivery (cells/µl)**	10840 (8935, 13835)	11350 (9110, 13050)	11200 (9013, 13398)	0.713
**Female gender**	12 (40.0%)	22 (46.8%)	34 (44.2%)	0.557
**Birth Weight (g)**	3080 (2945, 3325)	2950 (2800, 3268)	3000 (2800, 3280)	0.310
**Birth length (cm)**	47 (46, 48)	47 (44-48)	47 (45-48)	0.482
**Placental malaria**	9 (36.0%)	14 (37.8%)	23 (37.1%)	0.883
**Height at 12 weeks (cm)**	59 (58-60)	56 (55-59)	58 (55-59)	0.037
**Weight at 12 weeks (kg)**	5.69 (5.40, 6.76)	5.53 (5.00, 6.02)	5.55 (5.28, 6.14)	0.088
**Length-for-age Z score at 12 weeks**	-0.42 (-1.34, -0.13)	-2.01 (-2.88, -0.47)	-1.34 (-2.31, -0.360	0.037
**Weight-for-age Z score at 12 weeks**	-0.35 (-0.72, 0.73)	-0.42 (-1.28, -0.05)	-0.39 (-1.02, 0.10)	0.159
**Stunting at 12 weeks**	1 (9.1%)	11 (52.4%)	12 (37.5%)	0.016
**Underweight at 12 weeks**	0 (0.0%)	2 (9.5%)	2 (6.2%)	0.290

IQR=Interquartile range. Length- and Weight-for-age Z scores determined from WHO reference standards. P values are reported for chi-square tests of proportions for categorical variables [displayed as n (%)], and Mann-Whitney U tests for continuous variables [displayed as median, (IQR)].

Figure S1. Comparison of Spike antibody levels as measured by Luminex vs MSD

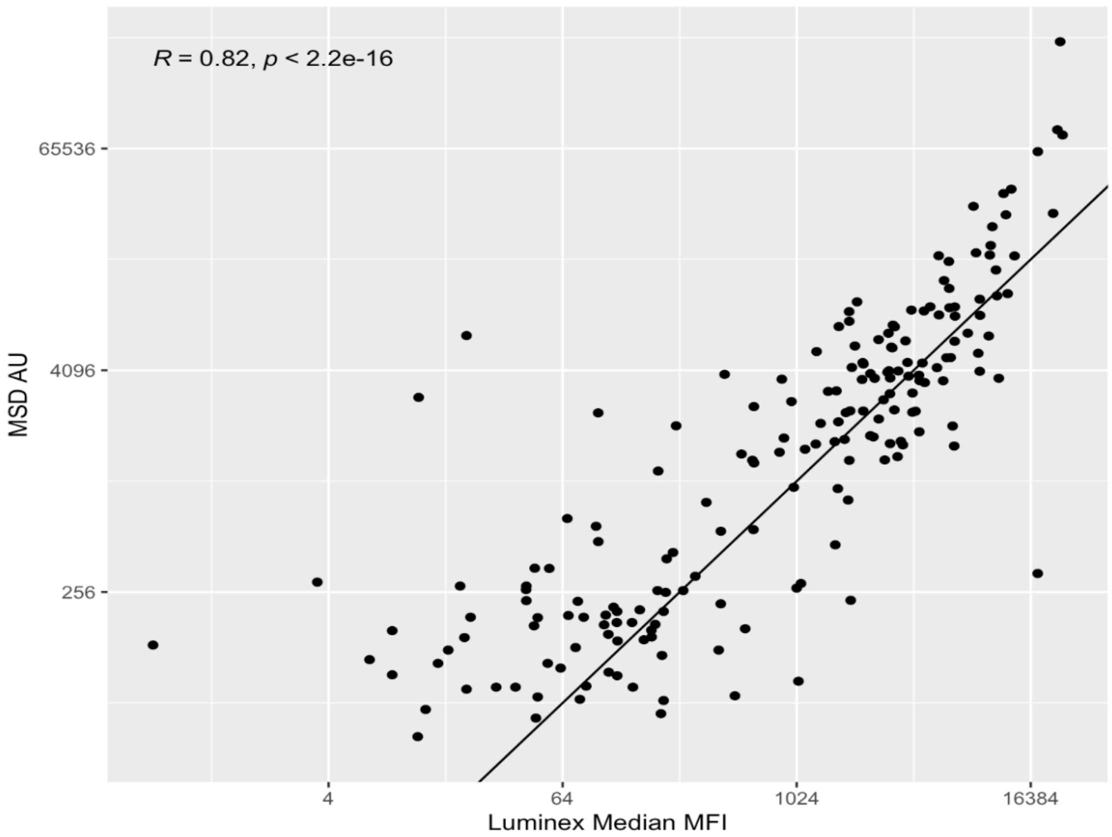

Correlation of Luminex SARS-CoV-2 Spike IgG median MFI with SARS-CoV-2 Wuhan-Hu-1 Spike IgG AU as measured on MSD platform.

Figure S2. Classification of SARS-CoV-2 During Pregnancy.

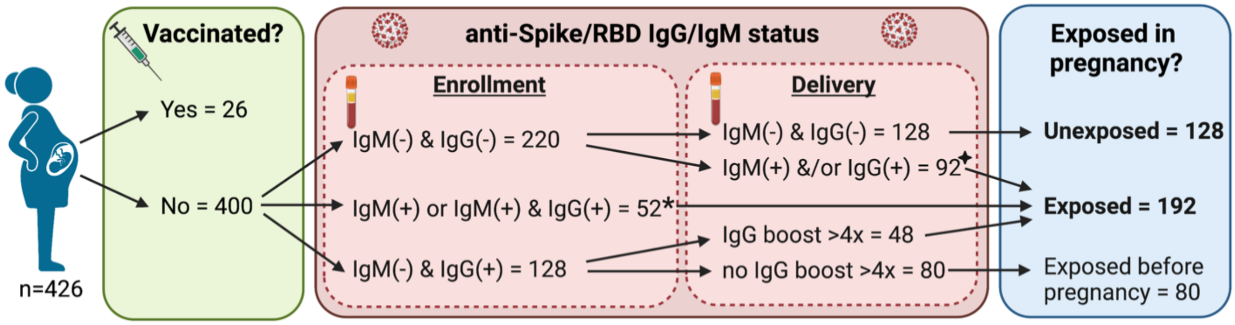

N=426 participants enrolled in a trial of malaria chemoprophylaxis in pregnancy in Uganda between December 2020 and February 2022 had stored plasma samples collected at enrollment (between 12 and 20 weeks gestation) and at time of delivery tested for SARS-CoV-2 Spike antigen and receptor binding domain (RBD) IgG and IgM using the MesoScale Discovery (MSD) platform. Of n=400 participants who remained unvaccinated to SARS-CoV-2 throughout study participation, 220 were negative for IgG and IgM to both Spike and RBD at enrollment. Of these, 128 remained negative at delivery and were presumed to have not been exposed to SARS-CoV-2 in pregnancy, and 92 were positive at delivery and were classified as seroconverted indicating SARS-CoV-2 infection during late✦ pregnancy. Of the 180 who were positive for IgG and/or IgM at enrollment, 128 were positive for IgG only, 12 for IgM only, and 40 for both IgG and IgM. The 52 who were positive for IgM only or IgM and IgG were presumed to have been infected with SARS-CoV-2 early* in pregnancy prior to study enrollment. Of the 128 who were positive for only IgG at enrollment, 48 had IgG antibodies boosted by a factor of 4 or greater at delivery and were presumed to have been re-infected during study participation. N=80 had only IgG positive at enrollment with negative IgM and no boosting at time of delivery, thus infection occurred prior to pregnancy or shortly thereafter, and timing is unable to be determined. Therefore, these participants were excluded from birth and infant outcome analysis.

Figure S3. SARS-CoV-2 surges in Uganda associated with symptoms and seropositivity at in the pregnancy cohort over time.

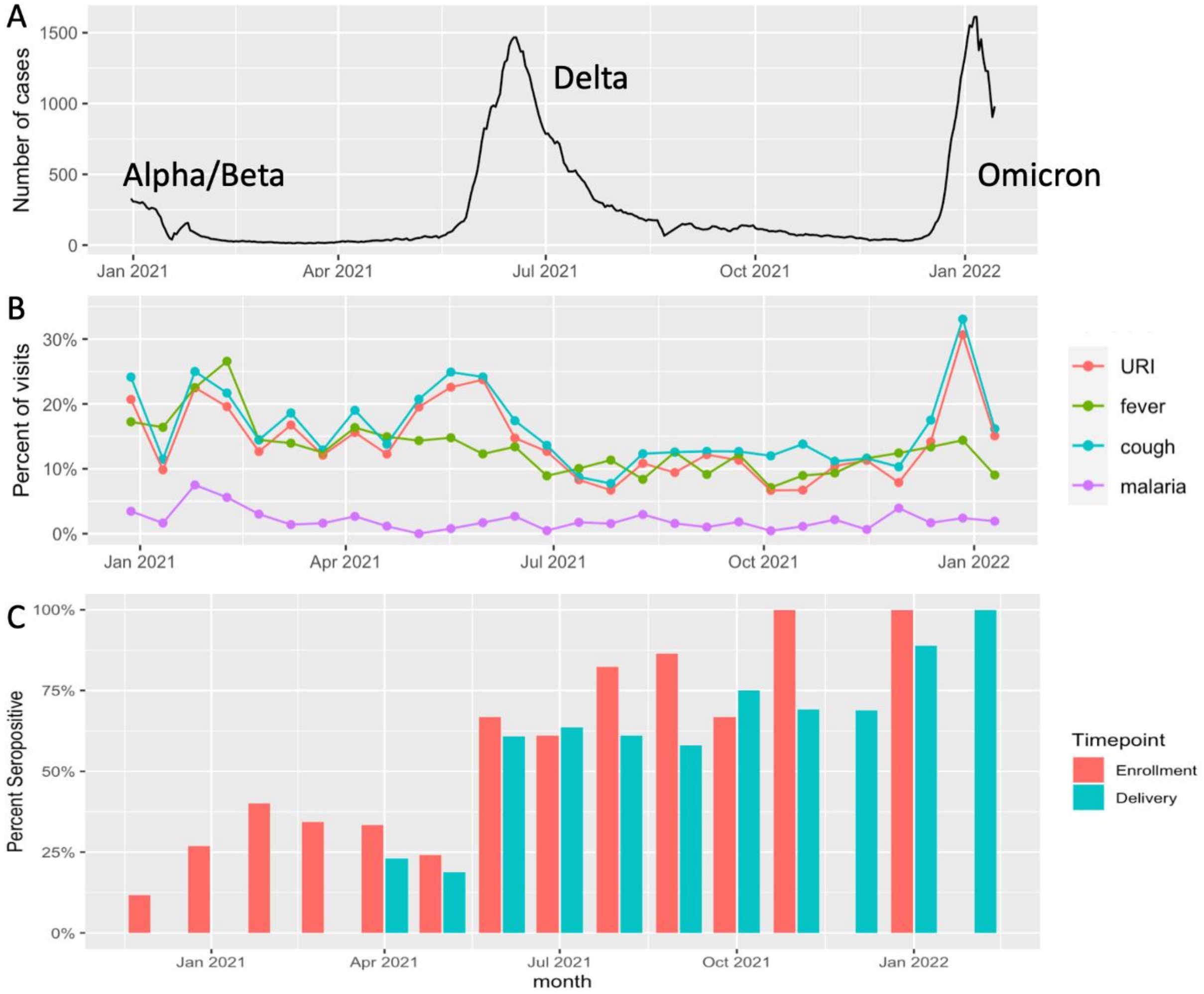

A. 7-day rolling average of daily new COVID-19 cases reported in Uganda (obtained from Our World in Data database, https://ourworldindata.org/coronavirus/country/uganda). One week in late August 2021 when >21,000 cases were reported in a single day is omitted. Surges are labeled with associated SARS-CoV-2 variants. B. Percent of study visits in each 2-week period from December 2020-February 2022 in which participants reported upper respiratory tract infection (URI), fever, cough, or were diagnosed with malaria (fever+positive blood smear with Plasmodium falciparum). Numbers of visits with reported cough or upper respiratory infections (URI) increased during the Delta and Omicron waves in Uganda. C. The proportion of seropositive tests at either enrollment (red) or delivery (blue) visits in each month increased from 12% in December 2020 to 100% in February 2022.

Figure S4. Variant of infection and association with infant growth through age 24 weeks

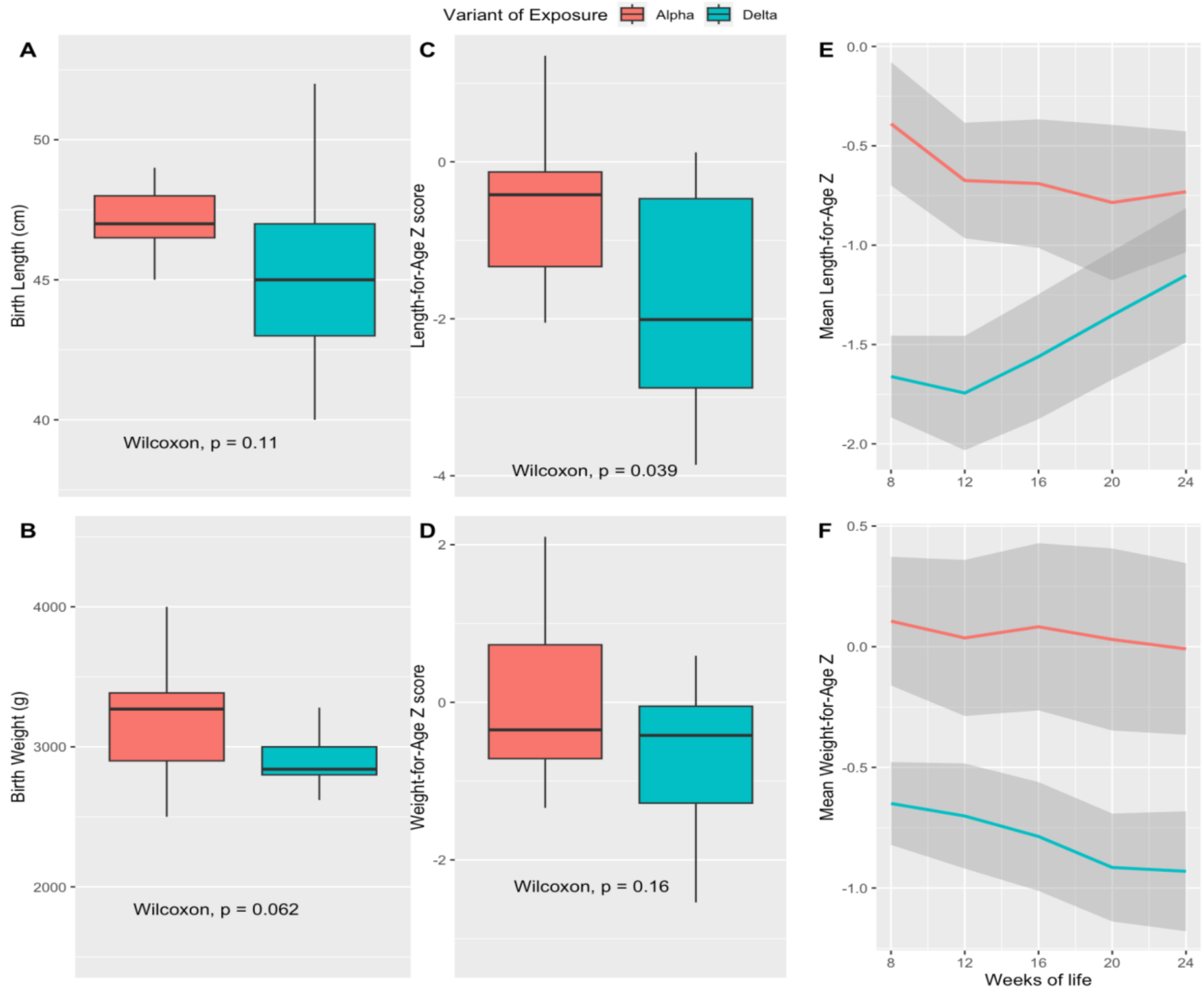

Length- and Weight-for-age Z scores determined from the WHO Child Growth reference standards. Boxplots revealing infant length at birth, infant weight at birth, length-for-age Z score at age 12 weeks, and length-for-age Z score at age 12 weeks comparing infants born to mothers exposed to SARS-CoV-2 Alpha versus Delta variant (A-D) and mean and 95% CIs (grey shaded region) of growth Z scores from 8 to 24 weeks of life (E-F). Alpha=Exposed mother with Wuhan-Hu-1:alpha variant RBD IgG AU ratio<1 and lowest of all variant ratios. Delta=Exposed mother with Wuhan-Hu-1:delta variant RBD IgG AU ratio<1 and lowest of all variant ratios.
